# Evaluation of Probiotic Metabolites Mediated Antimicrobial Activity and Inhibition of Virulence Factors in Avian Pathogenic *Escherichia Coli*

**DOI:** 10.1007/s12602-026-11027-5

**Published:** 2026-04-22

**Authors:** Kanchan Thapa, Anna Phan, Chuan-Wei Tung, Muhammad Abrar Hashmi, Debabrata Biswas

**Affiliations:** 1 Department of Animal and Avian Sciences, University of Maryland, College Park, MD 20742 USA; 2 Biological Sciences Program, Molecular and Cellular Biology, University of Maryland, College Park, MD 20742 USA

**Keywords:** Colibacillosis, Lactobacillus, Antimicrobial metabolite, Virulence attenuation, Antibiotic alternatives

## Abstract

Avian pathogenic *Escherichia coli* (APEC), a major etiological agent of colibacillosis in poultry, threatens both poultry industry and public health due to its economic impact, zoonotic potential, and rising multidrug resistance. As a safer and sustainable alternative to antibiotics, this study investigated the antagonistic effects of probiotics, specifically *Lactiplantibacillus plantarum* (LP) and *Lacticaseibacillus rhamnosus* (LR), against APEC growth and pathogenesis. Co-culture of APEC with either LP or LR at various ratios (1:1, 1:10) revealed a more rapid APEC inhibition corresponding to higher probiotics concentration with significant suppression within 24 h. The primary mechanism behind this inhibition was attributed to media acidification by LP and LR, as confirmed by pH measurements. Cell-free culture supernatants (CFCSs) collected from either LP or LR culture at both 24 and 48 h demonstrated time dependent bactericidal activity. CFCSs also significantly inhibited biofilm formation by > 3 log CFU/mL (*p* < 0.05) and disrupted pre-established APEC biofilms by > 2 log CFU/mL (*p* < 0.05). Gene expression analyses revealed marked downregulation of genes involved in adhesion, survival, membrane integrity and metabolic adaptation indicating attenuation of APEC virulence. Similarly, fluorescence microscopy analyses along with membrane permeability assays corroborated the disruption of bacterial membrane integrity due to CFCSs treatment. Together, our study demonstrates that probiotic-based strategies can be an alternative antibiotic-free intervention to prevent and control APEC infection in poultry.

## Introduction

Avian pathogenic *Escherichia coli* (APEC) represents an extra-intestinal pathotype of *E. coli* that causes significant morbidity and mortality in poultry, leading to considerable economic losses in the global poultry industry [1, 2]. APEC manifests itself with various clinical signs involving respiratory, systemic and reproductive issues such as airsacculitis, colisepticemia, perihepatitis, pericarditis, peritonitis, salpingitis cellulitis, omphalitis and osteomyelitis, the conditions collectively termed colibacillosis [3, 4]. Colibacillosis is frequently associated with serotypes O1, O2 and O78 however recent genome analyses have shown ongoing emergence of new highly virulent serogroups with antigenic diversity [5–11]. It is estimated that a minimum of 30% of commercial poultry flocks are affected by APEC at any given time in the US, with roughly $40 million annual losses due to carcass condemnation alone [4, 12]. Despite the recognized impact of APEC on commercial poultry, comprehensive and updated estimates of its full economic impact in the US remain lacking [13]. Beyond the substantial economic burden, this pathogen poses a food safety threat to global poultry products. Comparative genomics indicate that certain APEC lineages share substantial virulence and resistance genes with extra-intestinal pathogenic *E. coli* (ExPEC) isolates implicated in human infections, raising concerns that poultry may serve as a reservoir for zoonotic ExPEC and for antimicrobial resistance determinants [14–20]. These findings underscore the significance of APEC as a foodborne and zoonotic pathogen of public health relevance.

Conventional strategies for controlling colonization of APEC in poultry typically involve the use of antibiotics and vaccination along with implementation of stringent biosecurity measures [13]. However, following regulatory restrictions on the use of antibiotics as growth promoters or prophylactic agents in livestock particularly in the European Union and the US, concerns have emerged regarding decreased productivity and increased susceptibility of poultry to infectious agents such as APEC [20]. Although vaccines represent an important control measure, their efficacy is limited by the genetic diversity of APEC strains, which often results in suboptimal protective outcomes, consequently no ideal vaccine has been developed against APEC till date [21, 22]. These limitations reinforce the urgent need to develop alternative, sustainable, and safe strategies to mitigate APEC infections [20, 23, 24]. Currently, some effective alternatives under investigation include bacteriophages, essential oils, phytochemicals, prebiotics and probiotics [21–25]. Among these, probiotics and their metabolites have been one of the most promising avenues. Probiotics such as *Lactiplantibacillus plantarum* (LP), *Lacticaseibacillus rhamnosus* (LR), and several other *Lactobacillus spp.* along with their bioactive metabolites have shown numerous evidence against foodborne and zoonotic pathogens such as *Salmonella*, Enterohemorrhagic *E.coli*, *Listeria*, *Campylobacter spp*., *Staphylococcus spp.* among many others [26–29].

LP and LR have been shown to enhance host immunity and control respiratory pathogens such as *Streptococcus pneumoniae*, *Klebsiella pneumoniae*, *Pseudomonas aeruginosa* and *E. coli* through immunomodulatory and antimicrobial mechanisms [30–36]. Metabolites of these probiotics include diverse bioactive antimicrobial compounds, including organic acids, short-chain fatty acids (SCFAs), exopolysaccharides, bacteriocins, hydrogen peroxide and enzymes [37–39]. Mechanistically probiotic or their metabolites employ direct antimicrobial effects by membrane permeabilization or promoting pathogen cell lysis. Additionally, exopolysaccharides, probiotic cell wall components, SCFAs, etc. have been proven to mediate host immunomodulation by reinforcing epithelial barrier function or suppressing inflammatory pathways [35, 40–44]. While probiotics have been extensively studied for their beneficial effects on gut health in both poultry and humans, there is growing interest in their role in modulating respiratory infections [45, 46]. Of particular interest is the effectiveness of oral administered probiotics against respiratory infections, supporting the emerging concept of gut-lung axis-mediated immune modulation [47–51].

Recent studies have explored the modulation of lung microbial communities through oral probiotic administration as a potential preventive or immune stimulation strategy against APEC infection [52–54]. Most current research emphasizes probiotic supplementation for reducing disease severity, showing promising results, yet the underlying mechanisms remain poorly understood [27, 34, 38, 55, 56]. This study aims to investigate the concentration-dependent antagonistic activity of two well-recognized probiotic strains, LP and LR against APEC growth and virulence. Given their strain-specific antimicrobial and immunobiotic properties, evaluating underexplored strains of LP and LR against APEC is a critical step toward developing probiotic-based interventions for avian respiratory health. So, this study seeks to assess their potential to modulate the virulent characteristics of the pathogen, thereby contributing to a better understanding of probiotic-pathogen interactions in the context of avian respiratory disease.

## Materials and Methods

### Bacterial Strains, Culture Conditions and Media Compositions

The bacterial pathogen, Avian Pathogenic *E. coli* O78 (APEC) (Donated by Dr. Subhashinie Kariyawasam, Pennsylvania State University, US) was used in this study. APEC was grown on MacConkey agar (BD Biosciences, San Jose, CA, US) or in Brain Heart Infusion broth (BHI) (BD Biosciences, San Hose, CA, US) with shaking at 150 rpm for routine propagation at 37 °C aerobically. Two probiotic species *Lactiplantibacillus plantarum* (ATCC 8014) and *Lacticaseibacillus rhamnosus* (ATCC 11443) were grown in de Man Rogosa Sharpe (MRS) broth (Hardy Diagnostics, Santa Maria, CA, US) or on MRS agar using a CO_2_ (5%) incubator at 37 °C (Thermo Fisher Scientific Inc., Waltham, MA, US).

To determine a suitable medium that could support growth of both APEC and the probiotic strains in a co-culture condition, growth patterns of APEC, LP and LR were individually evaluated in two different media by culturing at: (i) BHI/MRS broth alone, and (ii) a 1:1 mixture of BHI and MRS broth (v/v). For co-culture experiments, bacteria were incubated under aerobic and static conditions at 37 °C to provide uniform growth environment to all bacterial species. Growth was monitored by bacterial quantification using serial dilution and plate count on respective agar at various time points as described previously [29].

### Co-culture of Pathogen and Probiotics

APEC was co-cultured with varying concentrations of probiotics, either LP or LR, yielding pathogen-to-probiotic ratios of 1:1, 1:10, 1:100, and 1:1000. Higher probiotic-to-pathogen ratios were used to model commensal-dominant in vivo environment and to ensure adequate production of probiotic-derived antimicrobial metabolites under co-culture conditions. Co-cultures were carried out in 10 mL of a 1:1 (v/v) mixture of BHI and MRS broth, in which the pH was naturally stabilized at ~ 6 upon mixing. Overnight culture of APEC was adjusted to OD_600nm_ of 0.1 (~ 10^8^ CFU/mL) using which the initial pathogen load was fixed to ~ 10^5^ CFU/mL. Similarly, the OD_600nm_ of overnight culture of LP and LR was adjusted to 1.0 (~ 10^9^ CFU/mL) which was used to achieve 10^5^, 10^6^, 10^7^ and 10^8^ CFU/mL as required. OD_600nm_ measurements were used only for preliminary inoculum standardization following methods described previously [57, 58]. Co-cultures were incubated under aerobic conditions at 37 °C and samples were collected at 0, 6, 12, 24, 36, and 48 h. To observe the time-dependent growth or inhibition pattern, bacterial enumeration was performed via serial dilution in phosphate-buffered saline (PBS; pH 7.4). APEC and probiotic strains were enumerated using MacConkey agar and MRS agar, respectively, based on their established selectivity and absence of cross-growth under these conditions. In parallel, pH of the co-culture medium was measured at each time point using a calibrated pH meter (Mettler Toledo FiveEasy™/FiveGo™, Columbus, OH, US) to monitor probiotic-induced acidification. All the experiments were performed in triplicates or more repetitions.

### Collection of Cell Free Cultural Supernatants (CFCSs) from Probiotic Cultures and Determination of their Effects

Cell-free culture supernatants (CFCSs) were collected from both 24 h and 48 h cultures of LP or LR grown in MRS broth in the CO_2_ incubator at 37 °C. CFCSs were obtained at two distinct time points to evaluate the one with higher antimicrobial properties. These probiotic cultures were centrifuged at 4000 × *g* for 20 min, and the supernatants were filtered through a 0.22 μm membrane filter (VWR International, Radnor, PA, US) to obtain sterile CFCSs. The pH of the collected supernatants was measured and stored at 4 °C.

A standardized APEC suspension (10⁵ CFU/mL) was incubated in BHI broth supplemented with 20% (v/v) of CFCSs derived from both 24 h and 48 h either LP or LR culture. 20% (v/v) of CFCSs was chosen as a minimum inhibitory concentration necessary to achieve visible inhibition of APEC growth under the tested conditions. The viable APEC counts were determined at 0, 4, 8, 24, 48, and 72 h. Bacterial quantification was performed via serial dilution in PBS, followed by plating on MacConkey agar. All experiments were conducted at least in triplicate.

### Biofilm Formation and Dispersal Assay

Biofilm formation and dispersal assays were conducted following the method previously described by Merritt, et al. (2006) with some modifications [59]. In preliminary screening experiments, both LP and LR were evaluated for their effects against APEC and the data indicated no statistically significant differences between the CFCSs in terms of their inhibitory effects on APEC growth, biofilm formation, or membrane-associated responses (data not shown). Given this functional equivalence, only LP CFCSs were selected for subsequent experiments to avoid methodological redundancy and to ensure focus on a representative strain.

Briefly, to determine biofilm inhibition, 10 µL of APEC O78 suspension (10⁷ CFU/mL) was co-incubated with 2% glucose (v/v) in BHI broth and supplemented with varying concentrations (10%, 20%, and 30%, v/v) of probiotic CFCSs in a final volume of 1 mL/well in a 24-well tissue culture plate (Greiner Bio-One, Monroe, NC, US). Glucose supplementation was used to facilitate biofilm formation, as previously described for different strains of *E.coli* [60, 61]. After overnight incubation at 37 °C, planktonic cells were discarded, and wells were washed gently with sterile PBS to leave behind only adhered biofilm. The attached biofilm was then mechanically disrupted following the method described by Tabashsum, et al. (2019), resuspended in PBS, and viable bacterial numbers were assessed by serial dilution and plating [62]. For biofilm dispersal assay, APEC biofilms were developed for 24 h under the same conditions without CFCSs as described above. Following initial incubation, the spent broth was discarded, fresh BHI was added, and those preformed biofilms were treated with 10%, 20%, and 30% (v/v) of CFCSs. The plates were incubated again at 37 °C overnight, following which similar techniques as above were followed to get bacterial counts.

## RNA Extraction and cDNA Preparation

Extraction of bacterial RNA was performed following the method described previously [29]. Briefly, 1 ml of APEC O78 culture (10⁸ CFU/mL) treated overnight with 50% (v/v) of 48 h CFCSs collected from LP cultures was centrifuged at 14,000 × g for 15 min, after which the supernatant was discarded and the pellet was lysed in 1 mL of TRIzol^®^ reagent (Molecular Research Center Inc., Cincinnati, OH, US). The lysate was incubated at room temperature for 5 min, followed by the addition of 200 µL chloroform (VWR International, Radnor, PA, US). The resultant mixture was vortexed, then followed with a 3-minute incubation and centrifugation at 14,000 × g for 15 min. The aqueous phase was transferred to a fresh tube, mixed with 500 µL isopropanol (Fisher Scientific, Waltham, MA, US) incubated for 10 min, and centrifuged. Finally, the RNA pellet was washed with 75% ethanol, centrifuged at 7,500 × g for 5 min, air-dried, and resuspended in 50 µL RNase-free water. RNA quality and concentration were measured using a NanoDrop spectrophotometer (Thermo Fisher Scientific Inc., Waltham, MA, US). For cDNA synthesis, 1 µg RNA was reverse transcribed using the high-capacity cDNA reverse transcription kit (Applied Biosystems, Foster City, CA, US), following the manufacturer’s protocol. The thermal cycling conditions were set as follows: 25 °C for 10 min, 37 °C for 120 min, and 85 °C for 5 min to complete cDNA synthesis.

### Relative Gene Expression using qRT-PCR

Quantitative real-time PCR was conducted in a final reaction volume of 20 µL, comprising 2 µL of synthesized cDNA (100 ng), 2 µL each of forward and reverse primers (200 nM), 6 µL of RNase-free water, and 10 µL of PerfeCTa SYBR Green Fast Mix (Quanta Bio, Beverly, MA, US). Amplification was performed using the Eco Real-Time PCR System (Illumina, San Diego, CA, US) under the following thermal cycling conditions: an initial denaturation at 95 °C for 30 s, followed by 40 cycles of denaturation at 95 °C for 5 s, annealing at 55 °C for 15 s, and extension at 72 °C for 10 s. Gene expression levels were quantified using the comparative Ct (2^–ΔΔCt^) method as described by Livak and Schmittgen, 2001 [63]. GAPDH was used as the housekeeping gene to normalize the expression of target genes. All qRT-PCR reactions were performed in triplicate. Primer sequences used for amplifying selected APEC O78 genes are listed in Table 1.

**Table 1:** Real time PCR primers used in this study

Genes	Function	Primer sequence	References
GAPDH	Glyceraldehyde-3-phosphate dehydrogenase	F: CGGTACCGTTGAAGTGAAAGA R: ACTTCGTCCCATTTCAGGTTAG	[64]
*RpoS*	Stress response and division	F: CTCAACATACGCAACCTG R: GTCATCAACTGGCTTATCC	[21]
*ompA*	Outer membrane protein	F: TGACCGAAACGGTAGGAAAC R: GGAATACCAGTGGACCAACAA	[64]
*murD*	Peptidoglycan biosynthesis (UDP-N-acetylmuramoyl Alanine- D-glutamate ligase)	F: GCGTGGTTAATGCTGATGATG R: CCTGCTGATGATTCAGGTGATA	[64]
*radA*	DNA repair protein	F: TGAAGTCAGCAACCCTTCGG R: GAATCTCCACCAGCAACGGA	This study
*ftsZ*	Cell division protein	F: GAACGTGGACTTTGCAGACG R: CTTCCAGCAGCGGAGAAGAA	This study
*phoP*	Two component system regulator	F: CACAGGTCATTTCGCTCCC R: CTTTGCTCACCACTTTGCCA	This study
*ompC*	Outer membrane protein	F: CTACACCGGTGGTCTGAAATA R: CCTACGCGAGTTGCGTTATAG	[65]
*luxS*	Mediates synthesis of autoinducer AI-2	F: ACGAGTGCATCTGGTAAGTG R: CAATGGAAGACGTGCTGAAAG	[64]
*iss*	Serum survival protein	F: CGCTCTGGCAATGCTTATTAC R: GAAATGATGGGTGATGGTTTCC	[64]
*H-NS*	Stress response and biofilm formation	F: CCGAACGAACTGCTGAATAG R: TTACCTTGCTCATCCATTGC	[21]
*fimC*	Mediates the adherence to host	F: GCTGGCAGGTATCCTGATATTC R: TGCCCTGCCGGATAAATTAC	[64]
f*yuA*	Influences the iron uptake capacity	F: CTTCCCTTCCGGTTCGTTAATC R: GGTACAGCCCAAACACCATATC	[64]

### Fluorescent Imaging to Assess Membrane Disruption of APEC

Bacterial membrane integrity following treatment with LP CFCSs were assessed using fluorescence-based staining methods using a combination of SYTO-9 and FM 4–64 dye as described previously [66]. APEC cultures (OD₆₀₀ = 0.1) were treated with 50% (v/v) of 48 h LP CFCSs overnight at 37 °C with shaking at 150 rpm. APEC treated with 50% isopropyl alcohol served as the positive control while APEC treated with 50% (v/v) of MRS broth was used as negative control. Following incubation, cells were washed and resuspended in 500 µL saline, then stained with FM 4–64 (1 µg/mL) and SYTO-9 (5 µM) (L7012; Molecular Probes, Eugene, OR, US) for 30 min in the dark at room temperature. After staining, cells were centrifuged and resuspended in 50 µL saline. Following the staining process, a 4 µL aliquot of stained suspension was mounted on a glass coverslip (22 × 60 mm) overlaid with a thin layer of 1.2% agarose in 20% LB medium. Samples were visualized using a Zeiss LSM 980 Airyscan 2 confocal microscope with a 63× oil immersion objective.

### Membrane Permeability Assays

Membrane permeability assays including crystal violet (CV) uptake and propidium iodide (PI) uptake as described previously with some modifications [67, 68]. Briefly, APEC cultures (OD₆₀₀ = 0.1) were treated with 50% (v/v) of 48 h LP CFCSs at three different timepoints- 6 h, 24 h and 48 h with 37 °C with shaking at 150 rpm. Bacterial cells treated with isopropyl alcohol served as negative control. Following incubation, the cells were washed twice with PBS (pH = 7.4). For CV uptake assay, the cells were resuspended in 10 µg/ml of CV, incubated for 10 min at 37 °C and then centrifuged at 14,000 rpm for 10 min. Following that optical density (OD_590nm_) of the supernatant was recorded. The OD_590nm_ of the CV solution used in the assay was taken and it was considered 100%. The percentage of crystal violet uptake of all the samples was calculated using the following formula: (OD_CV_-OD_Sample_/OD_CV_ × 100). For PI uptake, the washed cells were resuspended in 1mL PBS along with 10µM PI and incubated for 30 min at 25 °C. The fluorescence of the dye was then monitored using a fluorescence spectrophotometer at an excitation wavelength of 535 nm and an emission wavelength of 617 nm.

### Statistical Analysis

Statistical analyses were performed in GraphPad Prism software (v.10.4.2, GraphPad Software, San Diego, CA, US). One-way or two-way analysis of variance (ANOVA) was performed followed by Tukey’s post multiple comparison tests, based upon the structure of the dataset, to evaluate whether each treatment was significantly different from the control for each measure point. Statistical significance was identified at *p* < 0.05.

## Results

### Preliminary Evaluation of Culture Media for Pathogen-probiotic Co-culture Compatibility

**Fig. 1:**
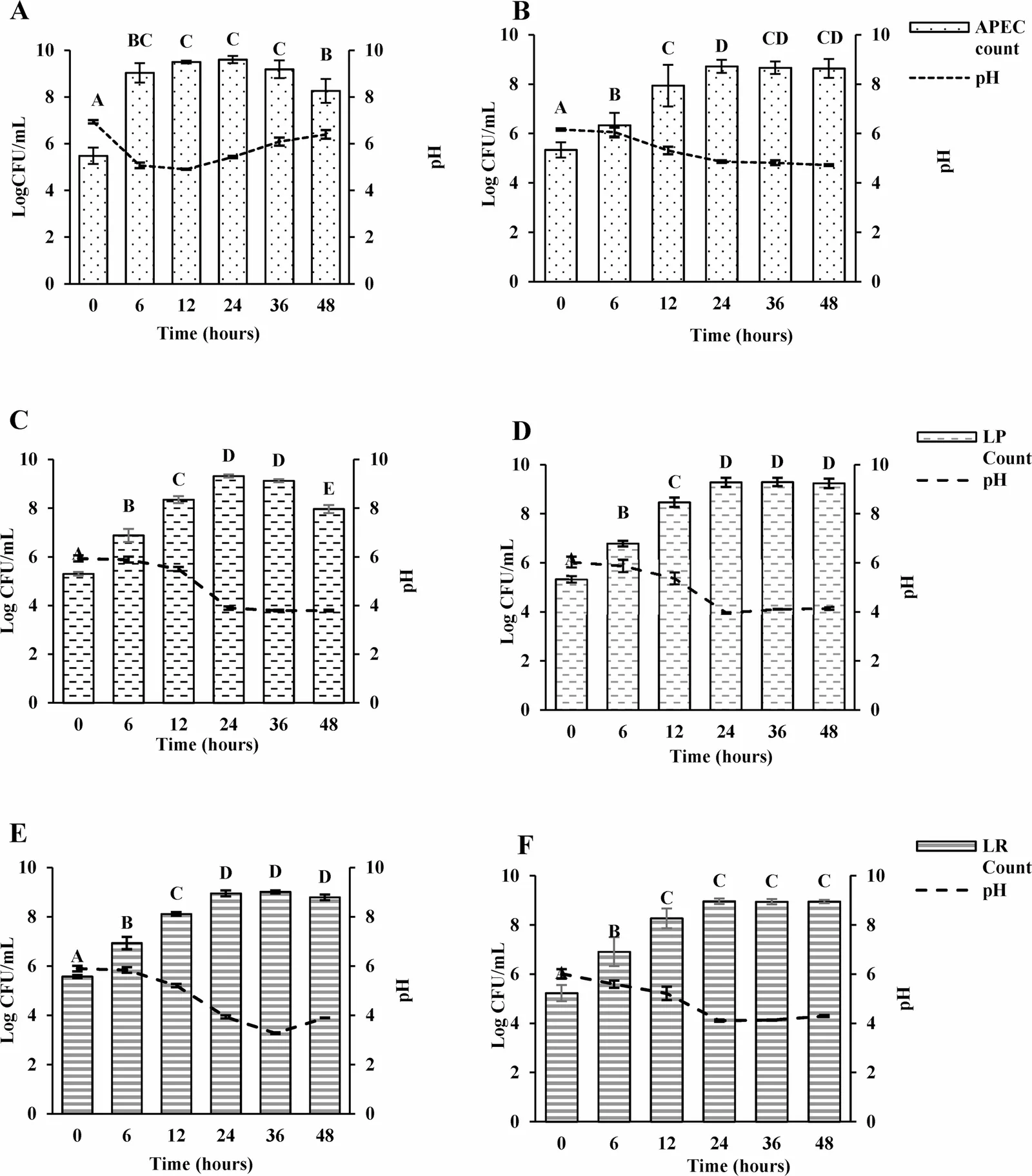
Time-dependent growth pattern of APEC in BHI (**A**), BHI-MRS combination (1:1, v/v) (**B**). Growth pattern of probiotics (LP, LR) in MRS (**C, E**), BHI-MRS combination (1:1, v/v) (**D, F**). A-D indicates significant differences between each of the groups. Dotted lines represent the pH changes at respective timepoints. *P* < 0.05, One-way ANOVA followed by Tukey’s post hoc test was followed.

To identify a suitable culture medium for the co-culture of APEC with either LP or LR, growth kinetics and pH changes were assessed in BHI only (for APEC) or MRS only (for LP and LR), and combination of MRS-BHI (1:1, v/v) media over a 48 h incubation period. APEC demonstrated optimal growth in BHI broth starting from an initial load of ~ 5.4 log CFU/mL, then increasing rapidly to ~ 9 log CFU/mL by 12 to 24 h, followed by a moderate decline to 8.2 ± 0.17 log CFU/mL at 48 h (Fig. 1A). The pH decreased from 6.9 ± 0.06 to 4.9 ± 0.04 at 12 h, then gradually increased to 6.3 ± 0.17 by 48 h, suggesting acid production during exponential growth and possible metabolic adaptation during the stationary phase. In the combination of MRS-BHI media, APEC showed a moderate reduction of growth until 12 h, then reached to > 8 log CFU/mL (Fig. 1B).

In MRS medium alone, both LP and LR showed rapid proliferation, increasing from an initial count of ~ 5 log CFU/mL to ~ 9 log CFU/mL at 24 h, followed by a decline at 48 h (Fig. 1C and E). A sharp decrease in pH from ~ 6 to ~ 4 by 24 h implied acid production by the probiotics. Growth in MRS-BHI combination medium was slightly more sustained, with LP (Fig. 1D) and LR (Fig. 1F) reaching ~ 9 log CFU/mL within 24 h and maintaining similar levels until 48 h. Statistically significant increases in CFU were noted from 12 h onward (*P* < 0.05), indicating the enhanced nutrient profile of the combined media supported both faster and prolonged growth.

### Exclusion of APEC by Probiotics

**Fig. 2:**
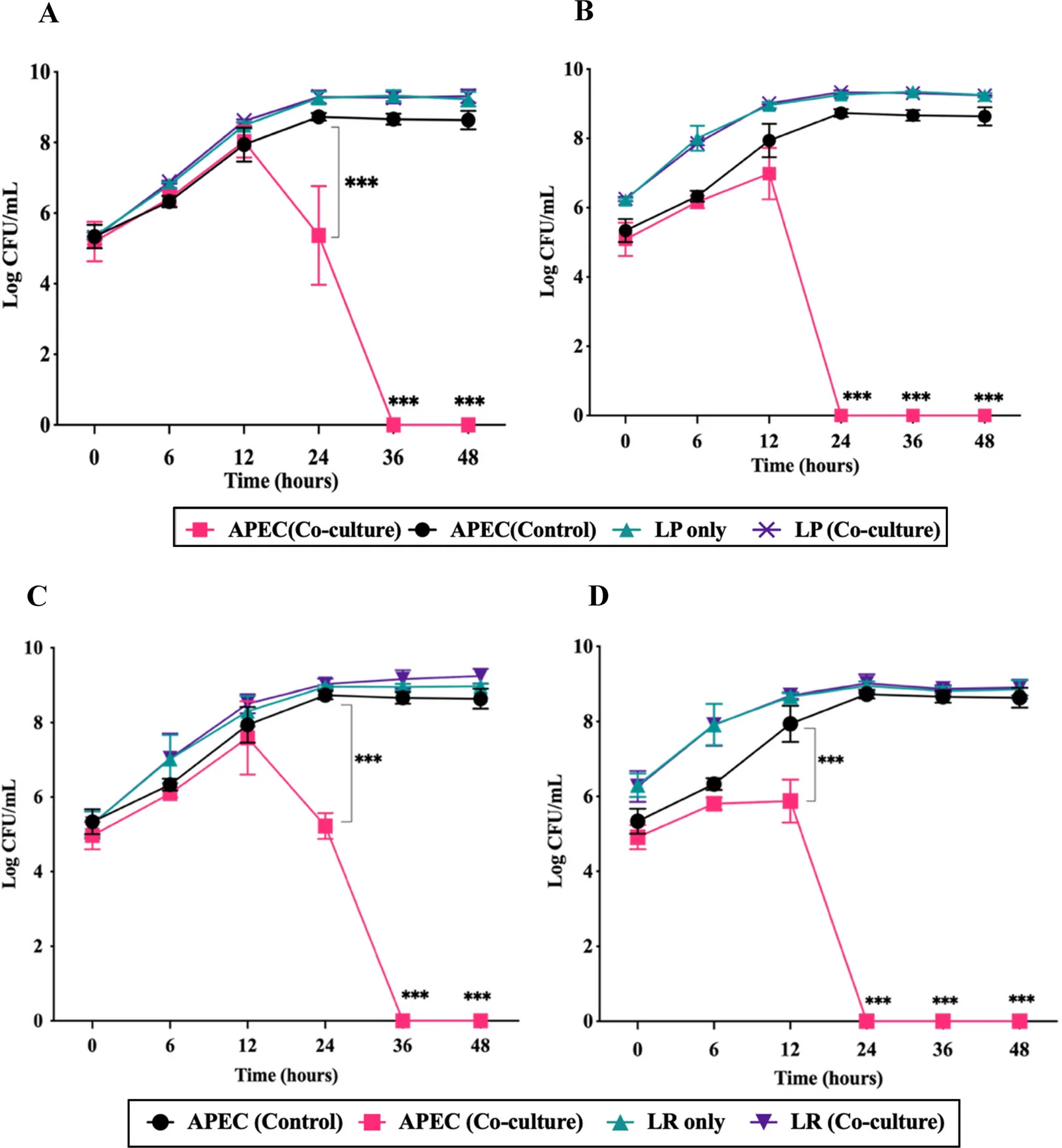
Growth inhibition of APEC in co-culture medium with LP at a pathogen-probiotic ratio of 1:1(**A**), 1:10(**B**) and with LR at a pathogen-probiotic ratio of 1:1(C), 1:10 (**D**). * represents significant difference of APEC in co-culture from the control group at each timepoint. * P<0.05, ** P<0.01 and *** P<0.001, two-way ANOVA followed by Tukey’s post hoc tests were followed

The inhibition potential of either LP or LR against APEC and associated pH changes of culture media was assessed by co-culturing at different APEC to probiotic ratios (Fig. 2). In all co-culture conditions, the initial APEC inoculum was standardized at ~ 10⁵ CFU/mL. APEC alone grew smoothly, reaching 8.6 ± 0.28 log CFU/mL by 24 h and maintaining ~ 8 log CFU/mL through 48 h across all conditions.

At a 1:1 ratio in APEC-LP co-culture, APEC showed no significant difference from control (APEC-only) until 12 h, whereas a significant reduction was observed at 24 h and onwards (*P* < 0.001) followed by complete elimination by 36 h of incubation (Fig. 2 A). In this same condition, pH of culture media declined sharply from 6.10 ± 0.07 at 0 h to 4.3 ± 0.04 by 24 h, aligning with the onset of APEC inhibition (Table 2). At a 1:10 ratio of APEC to LP, a similar trend was observed until 12 h, however it reached undetectable levels by 24 h, as compared to control (*P* < 0.001) (Fig. 2B). The pH of the co-culture media declined from 6.28 ± 0.04 to 4.14 ± 0.03, which suggests that acid production remained unchanged regardless of LP concentrations (Table 2).

**Table 2:** pH of the co-culture media with pathogen (APEC) and probiotic (LP or LR) at the ratios 1:1 and 1:10. Different letters ^(a-d)^ indicate significant differences within each probiotic group at each time-point

Time (hr)	APEC-LP Co-culture (Mean$\:\pm\:$SD)		APEC-LR Co-culture (Mean$\:\pm\:$SD)	
	1:1	1:10	1:1	1:10
0	6.10 ± 0.07	6.28 ± 0.04	6.11 ± 0.03	6.34 ± 0.03
6	6.10 ± 0.02ᵃ	6.17 ± 0.03ᵃ	5.72 ± 0.38ᵃ	5.82 ± 0.03ᵃ
12	5.08 ± 0.05ᵃ	4.70 ± 0.01ᵇᶜ	5.13 ± 0.38ᵃ	4.65 ± 0.02ᵇ
24	4.31 ± 0.05ᵃ	4.14 ± 0.03ᵃ	4.29 ± 0.08ᵃ	4.12 ± 0.03ᵃ
36	4.42 ± 0.03ᵃ	3.98 ± 0.02ᵃ	4.29 ± 0.14ᵃ	4.15 ± 0.03ᵃ
48	4.48 ± 0.15ᵃ	4.19 ± 0.02ᵃ	4.35 ± 0.02ᵃ	4.13 ± 0.04ᵃ

A similar pattern was observed in APEC-LR co-cultures where APEC initially increased to 7.34 ± 0.96 log CFU/mL by 12 h, then started to decline to 5.2 ± 0.4 log CFU/mL at 24 h followed by complete exclusion at 36 h (*P* < 0.001) (Fig. 2 C). This was accompanied by a pH drop from 6.11 ± 0.03 to ~ 4.29 by 24 h. (Table 2). Whereas, at a 1:10 ratio, APEC in co-culture increased remained consistent around 5 log CFU/mL till 6 h with significant reduction (*p* < 0.001) observed at 12 h timepoint. Finally, at 24 h there was complete inhibition (*p* < 0.001) (Fig. 2D).

In all the conditions, both LP and LR grew consistently reaching > 8 log CFU/mL when cultured alone or in co-culture medium indicating that media combination or APEC did not impact their growth. For both the pathogen-probiotics co-culture, the ratio higher than 1:10 (i.e. 1:100 and 1:1000) yielded a similar inhibition as that observed at 1:10 pathogen-probiotic ratio (Data not shown). The enhanced inhibition at a higher probiotic concentration suggests a dose and pH dependent inhibition.

### Growth Inhibition of APEC Using Probiotics CFCSs

**Fig. 3:**
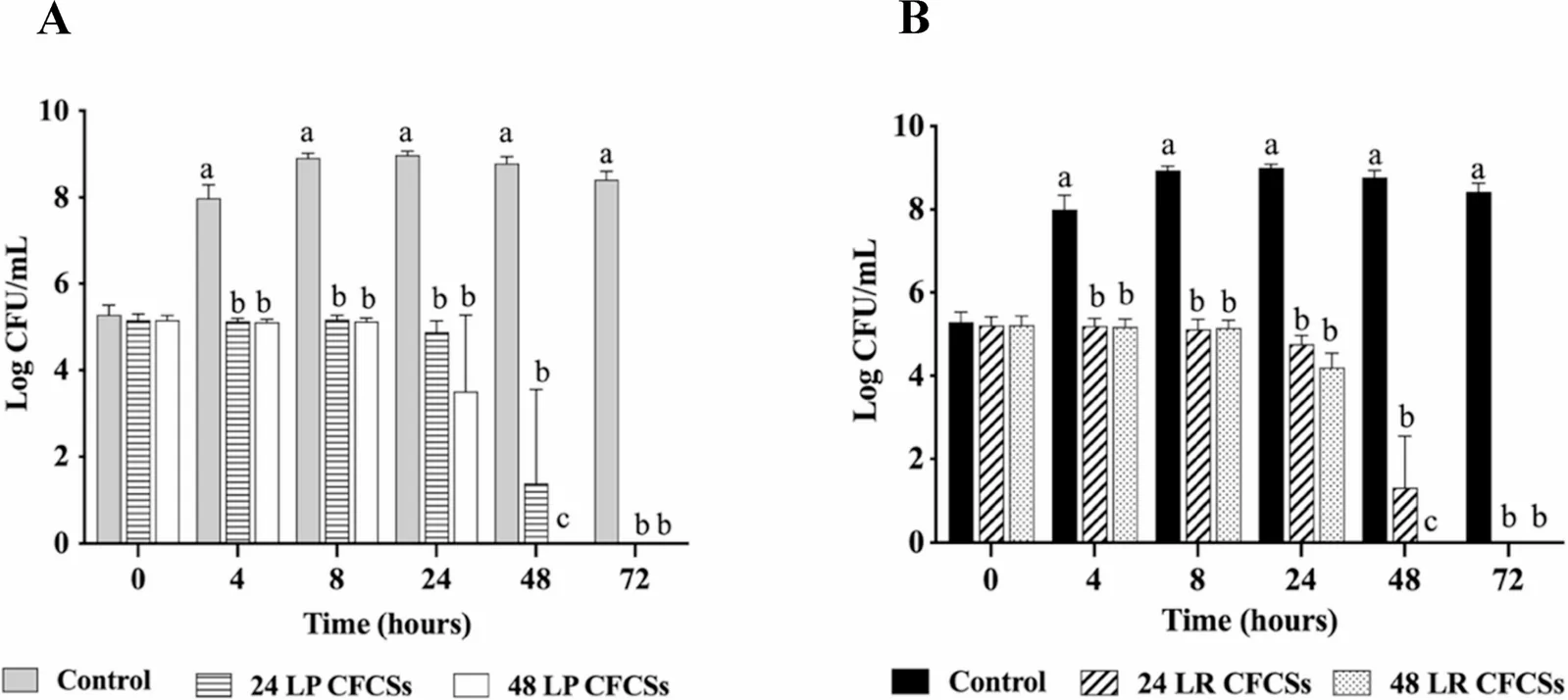
Time-dependent growth inhibition of APEC by CFCSs from 20% v/v LP (**A**) and 20% v/v LR (**B**). a-c denotes significant difference from the control group at each time point. P<0.05, two-way ANOVA followed by Tukey’s post hoc tests were followed

The inhibitory effects of CFCSs, collected from either LP or LR, against APEC were evaluated, with both supernatants showing original pH of approximately 3.9. Upon treatment with 20% (v/v) CFCSs from either LP or LR, APEC viability dropped significantly at all measured time points compared to the untreated controls (*P* < 0.05, Fig. 3). While control groups maintained consistent bacterial counts (approximately 8–9 log CFU/mL) throughout the 72 h period, those exposed to CFCSs showed minimal growth, with bacterial levels remaining near the initial inoculum (~ 5 log CFU/mL) up to 24 h post-treatment. By 72 h post-treatment, all tested CFCSs regardless of incubation periods or probiotic strain reduced APEC counts to undetectable levels. Notably, CFCSs collected after extended probiotic culture (48 h) exhibited stronger inhibitory activity against APEC compared to CFCSs collected at 24 h for both LP and LR (*P* < 0.05). This suggests that prolonged probiotic growth enhances the production and accumulation of antimicrobial metabolites, thereby increasing the potency of the resulting CFCSs.

### Inhibition and Dispersal of Biofilms by LP CFCSs

**Fig. 4:**
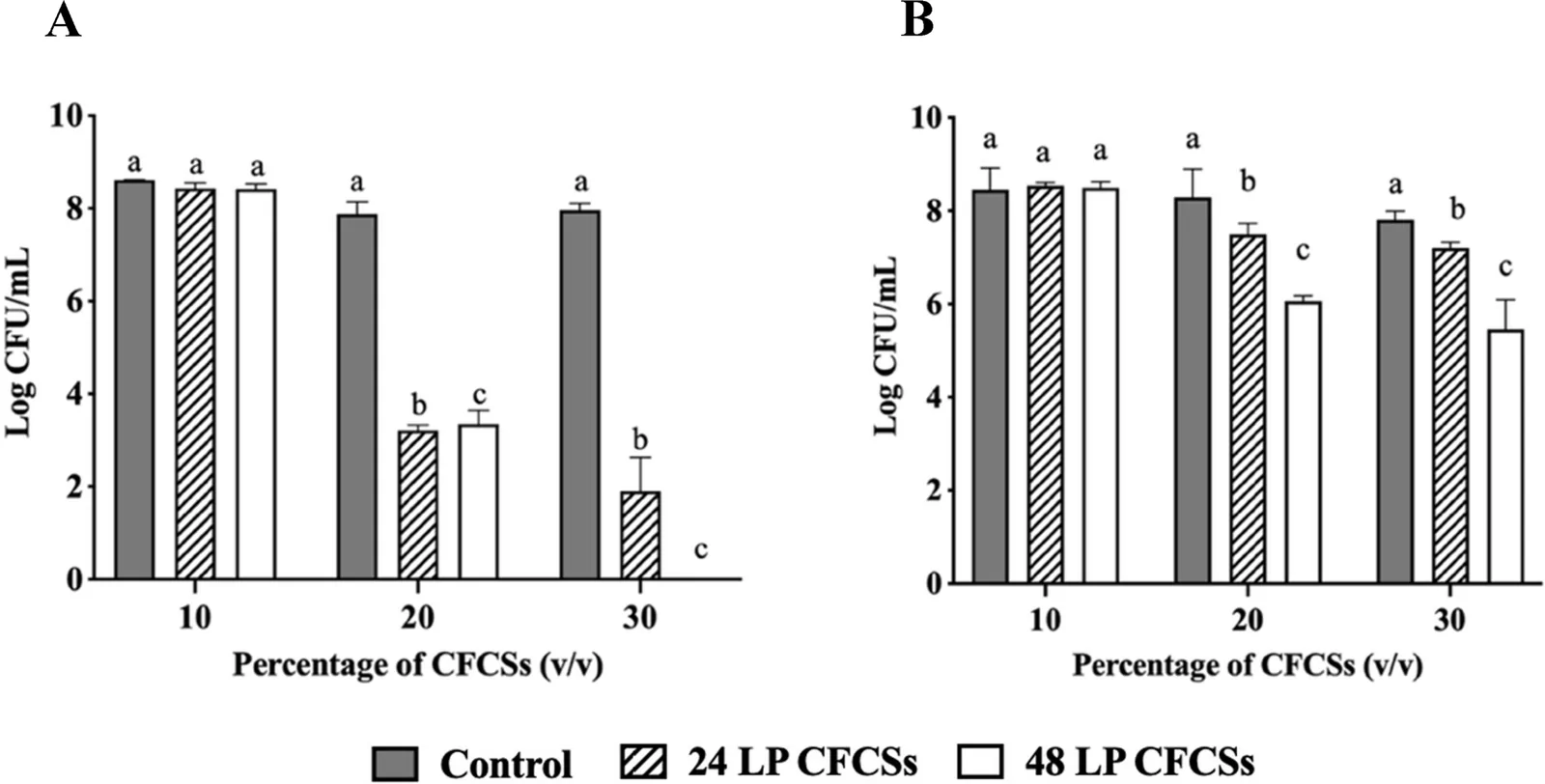
Biofilm inhibition of APEC by different concentrations of LP CFCSs (**A**) and biofilm dispersal of APEC by LP CFCSs (**B**). a-c denote significant difference from the control groups at each concentration of the CFCSs. P<0.05, two-way ANOVA followed by Tukey’s post hoc tests were followed

The inhibitory effects of CFCSs, collected from LP, on APEC biofilms were assessed at various concentrations- 10%, 20%, and 30% (v/v). Considering no significant differences between APEC co-cultures with either LP or LR, or in the inhibitory effects of their respective CFCSs, only the LP CFCSs was chosen for subsequent experiments. Concentration and time dependent biofilm-inhibitory activity of LP CFCSs against APEC biofilm formation were observed (Fig. 4 A). At concentrations of 20% and 30%, there was a significant reduction in the number of viable cells within the biofilm compared to untreated controls (*P* < 0.05). Notably, treatment with 48 h LP CFCSs at 30% led to the complete eradication of detectable APEC biofilm viability, whereas control groups maintained high bacterial counts at ~ 8 log CFU/mL (*P* < 0.05). Furthermore, the CFCSs preparations were also effective at disrupting pre-formed APEC biofilms. These mature biofilms initially exhibited higher bacterial loads than those seen in the inhibition assays, yet treatment with 48 h LP CFCSs at 30% resulted in a substantial decrease in biofilm biomass (Fig. 4B). Although the 10% concentration showed limited efficacy, higher concentrations produced statistically significant reductions (*P* < 0.05). Collectively, these findings underscore the potent anti-biofilm potential of LP CFCSs, with increased effectiveness observed at higher concentrations.

### Alteration of APEC Gene Expression by LP CFCSs

**Fig. 5:**
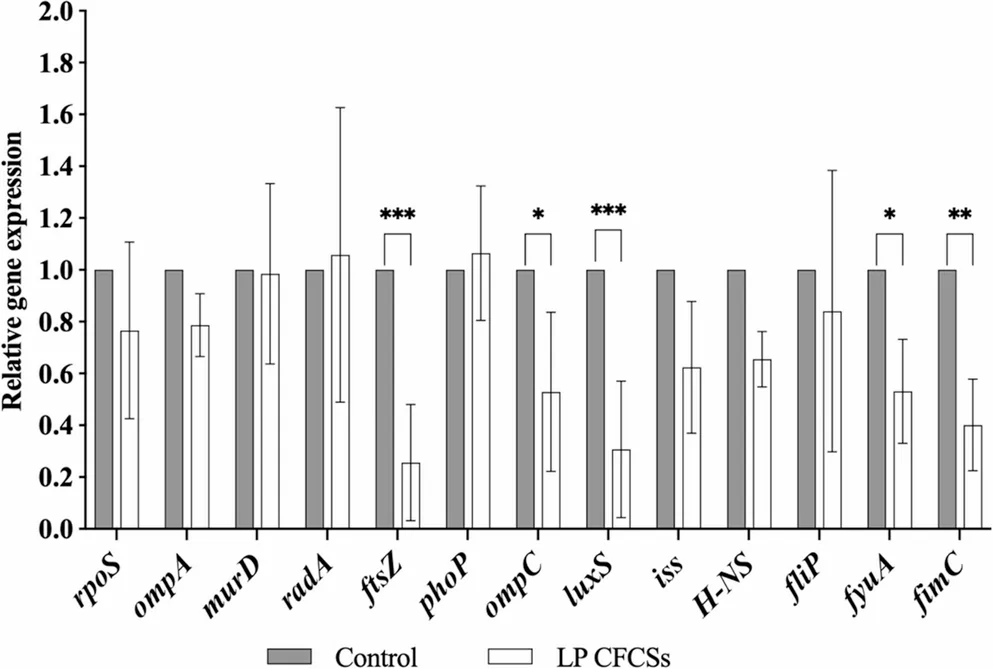
Relative expression of vital metabolic, survival and virulence-associated genes of APEC in response to LP CFCSs treatment compared to control. * represents significant difference from the control. Expression levels were normalized to GAPDH and presented relative to the untreated control, which was set to 1

To investigate the molecular basis of APEC inhibition, qRT-PCR was performed to determine the expression of key genes associated with survival, cell division, virulence, metabolism, and biofilm formation following exposure to 48 h CFCSs collected from LP. Gene expression levels were normalized to GAPDH and compared to untreated controls (Fig. 5). We observed downregulation of stress response genes (*rpoS*,* H-NS*) of APEC, although the changes were not statistically significant (*P* > 0.05). The outer membrane porin genes- *ompC* (*p* < 0.05) and *ompA* of APEC were downregulated while *murD*, a peptidoglycan biosynthesis associated gene; the metabolic regulator *phoP* and *radA-*a gene involved in DNA repair, showed no changes upon LP CFCSs exposure. The quorum sensing gene *luxS*, was significantly downregulated (*P* < 0.05), while *fliP* exhibited non-significant suppression. Finally, the iron acquisition gene *fyuA* and fimbrial chaperone protein *fimC* was significantly downregulated (*P* < 0.01) while serum resistance gene *iss* showed mild, non-significant downregulation upon LP CFCSs treatment. These findings suggest that probiotic-derived CFCSs impair APEC by disrupting key cellular functions, including division, structural maintenance, iron uptake, and virulence regulation.

### Observation of Membrane Alteration by Probiotic Metabolites

**Fig. 6:**
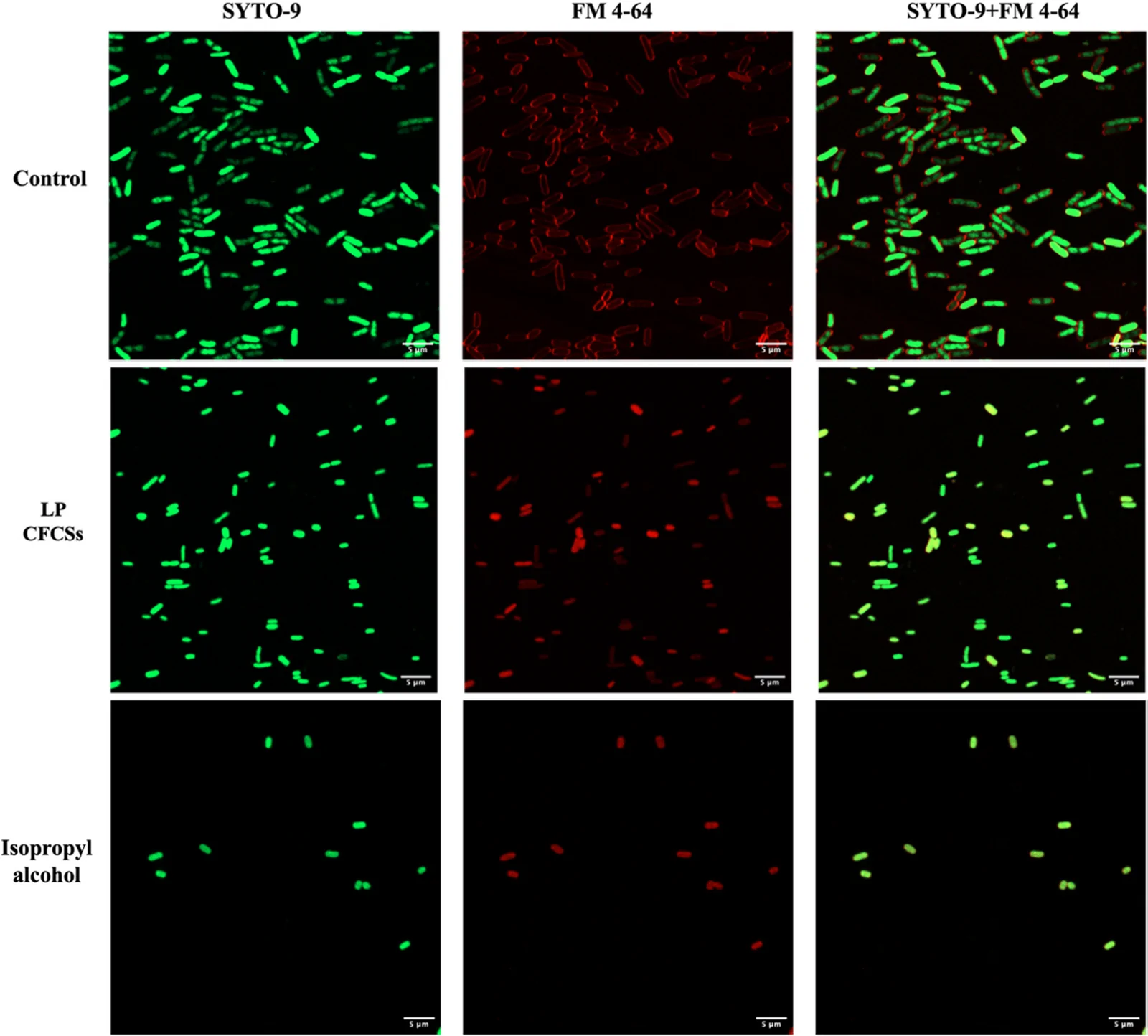
Fluorescence confocal microscopy images showing disrupted APEC membrane after 24 h of LP CFCSs treatment via SYTO-9 + FM4-64 staining. Green fluorescence (SYTO-9) represents the cytoplasmic content of bacteria and red fluorescence denotes the bacterial membrane and/or cytoplasm stained by FM4-64. All panels are at same scale (bar, 5 μm)

To investigate the effects of probiotic-derived metabolites on APEC membrane integrity, fluorescence microscopy was performed using SYTO-9 in combination with FM 4–64. In the SYTO‑9/FM 4‑64 assay, SYTO‑9 stains all bacterial membranes and emits green fluorescence when bound to cytoplasm, while FM 4‑64 is a lipophilic dye which inserts into intact outer membranes to produce a discrete red outline as observed in the control/untreated group (Fig. 6). However, in CFCSs treated and isopropanol treated positive control groups, FM 4–64 fluorescence became diffuse and colocalized with SYTO-9, indicative of compromised membrane architecture.

**Fig. 7:**
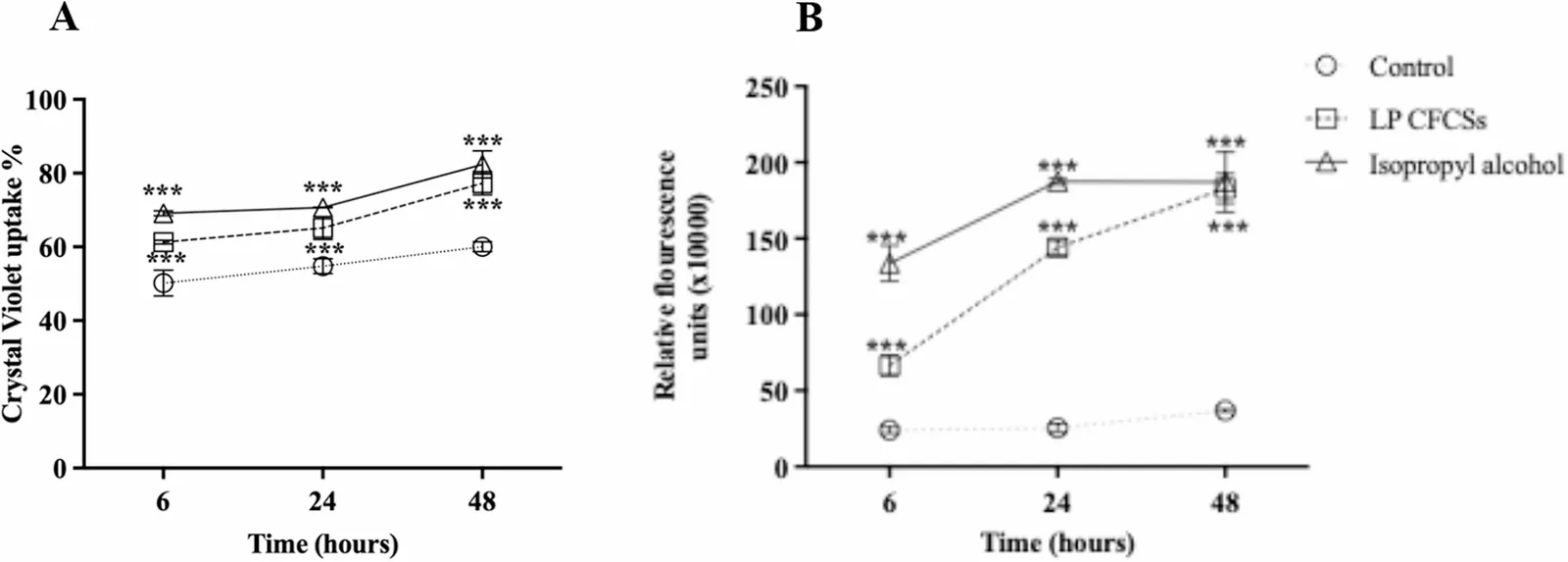
Membrane permeabilization of APEC by LP CFCSs shown through increased percentage of Crystal violet uptake by CFCSs treated cells (**A**) and increased relative fluorescence units (RFU) of Propidium Iodide with increasing treatment timepoints (**B**). * represents significant difference of each treatment group from the control (*p* < 0.001). Two-way ANOVA followed by Tukey’s post hoc tests were followed

In addition to this, the uptake of CV in LP CFCSs progressively increased at each of the timepoints measured which was significantly higher than the control group (Fig. 7A, *P* < 0.05). Similarly, the fluorescence intensity of PI was significantly higher in CFCSs treated bacterial cells than untreated control group at all timepoints suggesting alteration in membrane permeability (Fig. 7B, *P* < 0.05). These findings corroborate support a mode of action showing increase in membrane permeability by probiotic-derived metabolites, which could subsequently contribute to APEC cell death.

## Discussion

The increasing occurrence of multidrug-resistant APEC represents a dual threat to poultry industry sustainability as well as public health security, necessitating an urgent shift towards antibiotic-free alternatives. *Lactobacillus* spp. are widely recognized as promising probiotic candidates with extensive evidence supporting their ability to inhibit pathogenic colonization through multiple mechanisms such as competitive exclusion at adhesion sites, production of antimicrobial metabolites and modulation of host immune responses [34, 38, 39, 69, 70]. In this study we evaluated the potential of LP and LR as biocontrol agents against APEC by elucidating their antibacterial mechanisms through integrated approaches such as growth inhibition, biofilm disruption, gene expression analysis, and membrane integrity assessment.

Our co-culture experiments demonstrated dose-dependent antagonistic effects of both LP and LR (individually) against APEC. Higher initial probiotic concentrations (≥ 1:10 pathogen-to-probiotic ratio) achieved complete APEC elimination within 24 h, while lower ratios (1:1) delayed pathogen growth with complete suppression occurring by 36 h. This dose-dependent response reflects the combined effects of nutrient competition, adhesion site occupation, and rapid medium acidification. These findings align with established mechanisms whereby *Lactobacillus* species mediate pathogen inhibition through pH modulation and antimicrobial metabolite secretion [53, 54, 71, 72]. Moreover, the progressive pH decline observed during co-culture directly correlated with APEC population reduction, supporting organic acid production as a primary antimicrobial mechanism [37]. Multiple studies report the mode of action of undissociated organic acids, particularly lactic acid to be through the penetration of the gram-negative bacterial membranes, acidification of the cytoplasm, and disruption of membrane integrity ultimately resulting in pathogen death [30, 45]. These findings align with prior reports where *Lactobacillus* strains could inhibit pathogenic *E. coli* in poultry. For instance, Wang et al. (2017) showed that *L. plantarum* B1 reduced cecal *E. coli* counts and raised beneficial lactic acid bacteria populations [31]. Similarly, researchers also reported that *L. rhamnosus* GG demonstrated comparable efficacy against APEC through inhibition and competitive exclusion mechanisms [54]. Furthermore, studies have shown that lactate-producing *Lactobacillus* species contribute to the regulation of immune responses and support tissue regeneration within the intestinal mucosa making them promising candidates for both antimicrobial applications and the promotion of overall animal health [73, 74].

To expand upon these findings, we obtained the CFCSs from the probiotics to observe the direct inhibitory and bactericidal activity exhibited by metabolites secreted from either LP or LR. Exposure to 24 h CFCSs only arrested APEC proliferation whereas 48 h CFCSs completely eradicated the pathogen. This is consistent with previous reports showing that lactic acid bacteria, including LP and LR, increase production bioactive metabolites during late exponential and early stationary phases [75, 76]. The complete exclusion of APEC by 48 h in our study suggests that sufficient exposure to these antimicrobial metabolites beyond growth arrest leads to cell death. This observation aligns with prior studies where CFCSs from lactic acid bacteria drastically reduced *E. coli* populations over time [71, 77]. From these preliminary observations, both probiotics exhibited comparable anti-APEC effects, indicating functional redundancy between LP and LR in this context. However, LP was prioritized for subsequent assays because it is a predominant natural colonizer of the poultry gut [78, 79], with extensively documented immunomodulatory and antimicrobial activities, including emerging evidence supporting regulation of host defenses via the gut-lung axis [35, 36, 80–84]. Accordingly, only LP CFCSs were used in further experiments. Biofilm inhibition represents a critical virulence control mechanism, as APEC biofilms contribute significantly to treatment resistance and persistence in poultry production systems. CFCSs at concentrations ≥ 20% (v/v) significantly reduced developing APEC biofilm biomass corroborating previous reports where *Lactobacillus* have been found to secrete biosurfactants and signaling molecules that inhibit biofilm matrix synthesis [30]. For instance, Fernandes et al. (2012) showed that *Lactobacillus* CFCSs treatment of pre-formed *E. coli* biofilms decreased the residual biomass by ~ 50% [85]. Moreover, parallel to our findings, a cocktail of supernatants from variety of *Lactobacillus* significantly inhibited biofilms with slightly lesser dispersal of pre-formed biofilms against different gram-positive and gram-negative food-borne pathogens [42].

Gene expression analysis revealed comprehensive downregulation of key APEC genes, specifically virulence, stress response, and metabolic genes, during CFCSs treatment. Notably, the significant suppression of global regulators *H-NS* and *RpoS* indicates disruption of central regulatory networks governing bacterial stress adaptation and virulence [86, 87]. *H-NS*, a gene involved in nucleoid-associated protein, modulates transcription in response to environmental cues including pH and osmolarity; its suppression in response to CFCSs treatment suggests that probiotic metabolites may interfere with a central regulatory hub, weakening APEC’s stress tolerance and membrane defenses [86, 88, 89]. Similarly, *RpoS* downregulation indicates impaired stress response capacity, as this sigma factor governs adaptation to oxidative stress, nutrient limitation, and temperature fluctuations [87, 90]. The coordinated repression of these regulatory systems suggests that probiotic-derived compounds target primary bacterial survival mechanisms by impairing their capacity to mount effective stress responses, potentially increasing susceptibility to hostile conditions. Furthermore, the downregulation of specific virulence genes supports the diverse nature of probiotic antagonism. Suppression of *fimC* indicates reduced type 1 fimbriae assembly, potentially limiting APEC’s ability to adhere to respiratory and gastrointestinal epithelia [3, 91]. *Iss* (increased serum survival) is an important virulence-associated gene that enhances APEC’s resistance to the bactericidal action of serum complement so its reduced expression suggests diminished capacity to evade host complement-mediated killing [2]. Additionally, *ompA* and *ompC* (outer membrane porin) genes are responsible for passive diffusion of small molecules, nutrients, and antibiotics across the bacterial outer membrane expression, therefore their reduced expression might compromise membrane integrity and cellular transport functions of the pathogen [92].

The downregulation of *luxS*, which encodes the AI-2 synthase involved in quorum sensing, provides molecular evidence for biofilm inhibition mechanisms. *luxS*-mediated signaling regulates biofilm formation, motility, and virulence in enterobacteria, making it a critical target for pathogen control. The suppression of *luxS* gene in *E. coli* O157 by *L. acidophilus*-derived factors has been previously attributed to a quorum quenching mechanism [93]. The downregulation of this gene supports our biofilm inhibition results and implies that probiotic metabolites can disrupt bacterial communication systems. In addition, key genes involved in cell division-*ftsZ* was also downregulated, consistent with the bacteriostatic effect observed during the initial 24 h of CFCSs treatment. However, some genes that were not highly affected include *fliP*, involved in flagellar assembly; *radA*, associated with DNA repair; *murD* which is involved in peptidoglycan synthesis and *phoP*, a response regulator in the *PhoPQ* two-component system [94, 95]. Collectively, these transcriptional changes of some of the genes indicate disruption of APEC physiology, reinforcing the potential of probiotic CFCSs to impair bacterial virulence, adhesion, and survival which could be a synergistic effect of pH changes as well as multiple antimicrobial metabolites/peptides.

Furthermore, the use of lipophilic dye FM4-64 and SYTO-9 enabled us to visualize changes in membrane integrity. In intact Gram-negative bacteria, FM4-64, a lipophilic styryl dye, binds only the outer membrane surface forming a discrete red outline, so loss of this boundary indicates membrane breach which was apparent in the non-treated groups [57, 96]. However, upon treatment with probiotic CFCSs, instead of complete loss of the red outline, we observed a diffuse internal red fluorescence which implies that FM4-64 has penetrated past the outer leaflet and incorporated into the cytoplasm. This interpretation is supported by the bacterial studies with *Listeria monocytogenes* and *Salmonella enterica* co-staining experiments with FM4-64 along with labelled peptides [96]. This study visualized the similar appearance of stain patterns associated with FM4-64 which was taken as evidence of membrane disruption [96]. Thus, contrary to the expectation that FM4-64 would simply vanish when membranes are destroyed, its continued fluorescence throughout treated cells possibly indicates that membranes were leaky enough for the dye to enter. Moreover, the identical staining pattern seen with the isopropyl alcohol positive control supports our conclusion, as it is known to solubilize lipid bilayers producing membrane permeabilization [97]. Additionally, LP CFCSs exposure resulted in a significant, time-dependent increase in CV uptake, indicating perturbation of the APEC outer membrane. CV has limited ability to cross an intact outer membrane in enterobacteria but readily enters cells once the membrane is compromised [68, 98]. Consistent with this, PI fluorescence increased significantly in treated cells, suggesting loss of cytoplasmic membrane integrity. Because PI has been shown to penetrate only cells with compromised inner membranes, these findings indicate structural damage and membrane perturbation [67, 99]. Thus, the antibacterial activity observed following treatment with probiotic CFCSs may involve alterations in bacterial membrane permeability. Although the present study did not isolate or identify specific active compounds, or comprehensively assess all aspects of membrane disruption, the results are suggestive of possible membrane-associated effects. Given these limitations, future work will focus on the identification and characterization of individual probiotic metabolites and their precise contributions to membrane perturbation, including assays targeting intracellular leakage, membrane potential, and structural integrity.

## Conclusion

Overall, this study demonstrates that *Lactobacillus spp*. and their metabolites exert potent antagonistic activity against APEC through multiple in vitro mechanisms, including growth inhibition, biofilm suppression, membrane perturbation, and downregulation of key virulence-and stress associated genes. The results indicate that probiotic-derived metabolites, particularly those associated with acid-mediated effects play a central role in compromising APEC survival and pathogenicity. Importantly, these findings underscore the need for further identification and characterization of specific bioactive metabolites, as well as in vivo validation of immunomodulatory effects, to optimize probiotic formulations and advance targeted, antibiotic-free interventions for avian colibacillosis.

## Data Availability

The authors declare that the data supporting the findings of this study are available within the paper. Should any raw data files be needed in another format they are available from the corresponding author upon reasonable request.
